# Preoperative Carotid Duplex Scanning in Patients Undergoing Coronary Artery Bypass Grafting

**DOI:** 10.21470/1678-9741-2019-0131

**Published:** 2019

**Authors:** Hakan Kara

**Affiliations:** 1 Department of Cardiovascular Surgery, Giresun Ada Hospital, Giresun, Turkey.

**Keywords:** Coronary Artery Bypass, Carotid Endarterectomy, Carotid Stenosis, Risk, Factors, Stroke, Ultrasonography, Doppler, Duplex

## Abstract

**Objective:**

The aim of this study was to determine the prevalence and risk factors of carotid artery stenosis (CAS) using carotid duplex ultrasound in patients undergoing coronary artery bypass grafting (CABG).

**Methods:**

This retrospective study was conducted between January 2017 and January 2018 and included 166 consecutive patients [130 males (78.31%), 36 females (21.69%); mean age: 64.25±9.78 years] who underwent elective and isolated CABG. Patients who had significant CAS (≥50% stenosis) were compared with patients who had non-significant CAS (<50% stenosis). Logistic regression analysis was applied across the selected parameters to identify risk factors for significant CAS.

**Results:**

Of all patients, 36 (21.68%) had CAS ≥50% and 8 (4.81%) had unilateral carotid stenosis ≥70%. Carotid endarterectomy/CABG was performed simultaneously in five (3.01%) patients. None of these patients had cardiac and neurological problems during the postoperative period. The overall incidence of cerebrovascular accident (CVA) after CABG was 1.20% (n=2). Age (*P*=0.011) and history of CVA (*P*=0.035) were significantly higher in the CAS ≥50 group than in the CAS <50 group. Significant CAS was identified as a risk factor for postoperative CVA (*P*=0.013).

**Conclusion:**

Age and history of CVA were identified as risk factors for significant CAS. Furthermore, significant CAS was identified as a risk factor for postoperative CVA. For this reason, carotid screening is recommended for patients undergoing CABG even in the absence of associated risk factors.

**Table:** 

Abbreviations, acronyms & symbols				
ACAS	= Asymptomatic Carotid Artery Study		EACTS	= European Association for Cardio-Thoracic Surgery
ACT	= Activated clotting time		ESC	= European Society of Cardiology
AF	= Atrial fibrillation		ECST	= European Carotid Surgery Trial
ASA	= Acetylsalicylic acid		HT	= Hypertension
BMI	= Body mass index		ICA	= Internal carotid artery
CABG	= Coronary artery bypass grafting		LITA	= Left internal thoracic artery
CAS	= Carotid artery stenosis		LMCA	= Left main coronary artery
CCA	= Common carotid artery		NASCET	= North American Symptomatic Carotid Endarterec tomy Trial
CEA	= Carotid endarterectomy		OR	= Odds ratio
CI	= Confidence interval		PAD	= Peripheral arterial disease
CPB	= Cardiopulmonary bypass		SVG	= Saphenous vein graft
CVA	= Cerebrovascular accident		TIA	= Transient ischemic attack
DM	= Diabetes mellitus			
DUS	= Duplex ultrasound			

## INTRODUCTION

The coexistence of carotid and coronary artery diseases is an important pathology for cardiovascular surgeons. Carotid artery stenosis (CAS) is a significant risk factor for cerebrovascular accident (CVA) in cardiac surgery^[1]^. Preoperative screening and management of CAS in patients undergoing coronary artery bypass grafting (CABG) is important to reduce morbidity. It is unclear whether preoperative carotid screening should be applied to all patients^[2]^. Carotid duplex ultrasound (DUS) is the cheapest and most available method to accurately identify significant extracranial carotid stenosis^[3]^. Some centers perform preoperative carotid DUS on selected patients, while others perform it routinely^[4]^. North American Symptomatic Carotid Endarterectomy Trial (NASCET), European Carotid Surgery Trial (ECST), and the Asymptomatic Carotid Artery Study (ACAS) have reported that carotid endarterectomy (CEA) reduces the risk of CVA in symptomatic or asymptomatic patients with severe CAS^[5-7]^. In a meta-analysis, cardiac surgery patients with symptomatic/asymptomatic 50-99% stenosis or occlusion had a 7.4% CVA risk, which further increased to 9.1% in those with 80-99% stenosis or occlusion^[8]^. Based on these data, it can be said that among patients scheduled for CABG, it is highly important to identify patients with severe CAS in the preoperative period and treat them with CEA, and to take patients with moderate CAS into follow-up programs. The aim of this study was to determine the prevalence and risk factors of CAS using carotid DUS in patients undergoing CABG.

## METHODS

This retrospective study was performed on patients who underwent preoperative carotid DUS prior to scheduled elective primary isolated CABG between January 2017 and January 2018. The study included 166 consecutive patients [130 males (78.31%), 36 females (21.69%)], with a mean age of 64.25±9.78 years. Exclusion criteria included preoperative atrial fibrillation (AF), postoperative AF, antiarrhythmic treatment (other than beta-blockers), off-pump CABG, preoperative chronic obstructive pulmonary disease, emergency surgeries, redo surgery, bleeding and/or tamponade revision, chronic kidney failure, and combined (other than CEA) surgeries. The study was approved by the Ethics Review Committee of Karadeniz Technical University Faculty of Medicine (number: 31.12.2018/ 295). All protocols were in compliance with the ethical guidelines of the Declaration of Helsinki of 1975.

**Fig. 1:**
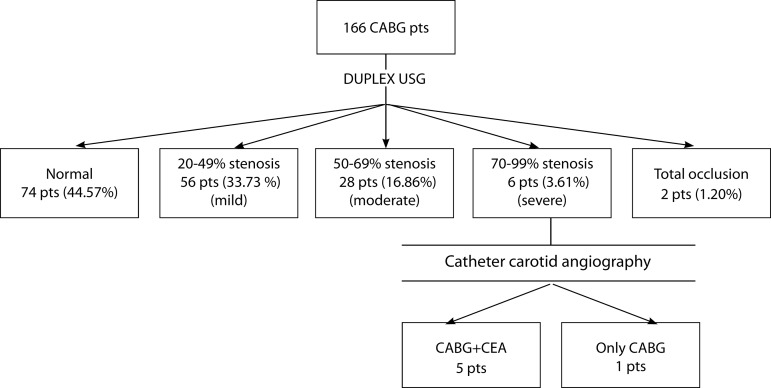
Outcome summary for the 166 CABG patients who underwent preoperative screening. CABG=coronary artery bypass grafting; CEA=carotid endarterectomy; DUS=Doppler ultrasound; pts=patients

Data collected included patient demographics, comorbidities, history of previous CVA, preoperative carotid artery duplex scan results, postoperative CVA, and postoperative details. Evaluation of internal carotid artery (ICA) stenosis was performed with DUS. The carotid DUS of all patients were performed by the same radiologist. Ultrasonic examination of the neck was performed using a commercial Doppler device (GE Logic S6, USA) with a linear probe 8.0-12.0 MHz to determine the presence of an atheroma or occlusion blood flow involving the carotid arteries bilaterally. Color Doppler was used to obtain blood flow velocities in the common (CCA), internal (ICA), and external carotid arteries. Parameters recorded were ICA peak systolic velocity, ICA/common carotid artery peak systolic velocity ratio and ICA end-diastolic velocity. The presence of plaque, calcification, and intimal thickening involving the carotid vessels was observed. A diagnosis of CAS made its severity graded according to the Society of Radiologists in Ultrasound criteria^[9]^. The standard protocol applied to all patients was based on the following criteria for defining a significant carotid stenosis: ICA peak systolic velocity of ≥250 cm/s, ICA end-diastolic velocity ≥120 cm/s, and velocity to the ICA (VICA)/velocity to the CCA (VCCA) of >1.8 to the systolic phase and >2.6 to the diastolic phase. Patients were classified into five groups as shown in Figure 1. A bruit may not be heard as the grade of stenosis in the carotid arteries increases. The presence or absence of a cervical bruit is a poor indicator of a high-grade carotid stenosis, even in the setting of known symptomatic disease (sensitivity 63%, specificity 61%)^[10]^. For this reason, bruit was not evaluated in our study during the preoperative evaluation. Patients with significant CAS (≥50% stenosis) and those with <50% stenosis were compared in terms of age, gender, diabetes mellitus (DM), hypertension (HT), dyslipidemia, body mass index (BMI), peripheral arterial disease (PAD), smoking, history of cerebrovascular accident (CVA), number of graft vessels, and left main coronary artery (LMCA) disease.

A routine preoperative evaluation was performed for each patient. In our study, other antiagregant and anticoagulant drugs excluded acetylsalicylic acid (ASA) were withdrawn before CABG. Patients with critical coronary artery disease or significant carotid artery stenosis (≥50% stenosis) were treated with ASA and low-molecular-weight heparin. Premedication, induction, and maintenance anesthesia, along with intraoperative hemodynamic monitoring and anticoagulation, were performed according to the standard protocol. Surgery was performed using median sternotomy. The left internal thoracic artery (LITA) and the saphenous vein graft (SVG) were prepared. Standard cardiopulmonary bypass (CPB) techniques (Stöckert S3 Sorin Group, Italy) with moderate systemic hypothermia (28-30°C) were employed. Myocardial preservation was achieved by antegrade blood cardioplegia for cardiac arrest and maintenance with antegrade and retrograde blood cardioplegia. Proximal anastomoses were performed with a single clamp. Prior to initiation of CPB, all patients received a standard heparin dosage of 300 IU/kg bodyweight, with an additional optional bolus of 50 IU/kg prior to cannulation. Anticoagulation was monitored by serial measurements of activated clotting time (ACT), which was kept > 450 s at all times during CPB. Heparin was reversed by administering 1 mL of protamine for each 1000 IU heparin, and ACT return to baseline served as confirmation. Carotid artery angiography was also performed in patients with severe stenosis identified by carotid DUS and planned for CEA. Angiographic and carotid DUS data were consistent with each other. CEA was performed under general anesthesia simultaneously with CABG. CEA was performed first. During CEA, the use of shunt was decided by assessing the retrograde flow in ICA. Shunt was used in one patient who was thought to have inadequate retrograde flow and whose stump pressure was measured as 25 mmHg and was not required in the other four patients. Because the ICA diameter was >4 mm, arteriotomy was primarily closed in all patients.

### Evaluation of Carotid Artery Stenosis

All patients scheduled for elective CABG surgery underwent routine preoperative duplex ultrasonography scanning of the carotid artery to assess for stenosis. The degree of stenosis was grouped into five categories: no stenosis, 20 to 49% (mild stenosis), 50 to 69% (moderate stenosis), ≥70% (severe stenosis), and total occlusion based on peak systolic and diastolic velocities (Figure 1). Our institution’s criteria for CEA prior to or concurrent with cardiac surgery is symptomatic patients with >50% stenosis or asymptomatic patients with >70% bilateral stenosis and asymptomatic patients with >80% unilateral stenosis.

### Postoperative Cerebrovascular Accident

A postoperative CVA or stroke was defined as a persistent focal or multifocal neurological deficit, explained as a brain or brainstem ischemia that occurs from the time of surgery until the 30^th^ postoperative day, and confirmed using magnetic resonance imaging. Neurocognitive dysfunction was not assessed in our study.

### Statistical Analysis

Clinical characteristics are presented as mean ± standard deviation for continuous variables and proportions for categorical variables. Data are presented as frequencies and percentages according to the type. Statistical analyses were conducted using Statistical Package for the Social Sciences (version 23.0; IBM Corporation, Armonk, NY, USA) software, and *P*<0.05 was considered statistically significant. Logistic regression analysis was used to obtain odds ratio (OR) and the corresponding 95% confidence interval (CI) to analyze risk factors.

## RESULTS

A total of 166 patients (130 males, 78.31%; 36 females, 21.69%; median age 64.25±9.78 years) underwent carotid artery DUS before undergoing CABG within the study period. Among the patients, 56 (33.73%) had 20-49% CAS, and 28 (16.86%) had 50-69% CAS. Of these, four (2.40%) had bilateral 50-69% CAS. Further, six (3.61%) patients had ≥ 70-99% CAS. In two (1.20%) patients, total occlusion was detected in any of the carotid arteries (Figure 1). Two (1.20%) patients underwent intervention in one of the carotid arteries before CABG. Furthermore, 34 (20.48%) patients had LMCA disease. Of the six patients with 70-99% CAS, five (3.01%) underwent simultaneous combined CEA/CABG, and four of these patients had unilateral 90% CAS and the remaining one had symptomatic 70% CAS. One patient with unilateral asymptomatic 70% stenosis underwent CABG only. The mean age of eight patients with ≥70% stenosis was 66.00±11.94 (range 54-82) years.

Demographic characteristics of patients with CAS ≥50% and CAS <50% were: age (68.66±9.46 *vs*. 63.04±9.55), male gender (n=29, 80.55% *vs*. n=101, 77.69%), DM (n=16, 44.44% *vs*. n=49, 37.69%), HT (n=30, 83.33% *vs*. n=96, 73.84%), dyslipidemia (n=19, 52.77% *vs*. n=68, 52.30%), BMI (26.83±2.92 *vs*. 26.90±3.38), history of PAD (n=6, 16.66% *vs*. n=11, 8.46%), smoking (n=27, 75.00% *vs*. n=96, 73.84%), history of CVA (n=5, 13.88% *vs*. n=2, 1.53%), EF (61.22±8.84 *vs*. 61.75±9.91), aortic cross-clamp time (75.50±18.46 *vs*. 76.11±23.66), total perfusion time (116.31±20.56 *vs*. 117.34±28.72), number of graft vessels (3.86±0.99 *vs*. 3.41±0.92), and LMCA disease (n=10, 27.77% *vs*. n=24, 18.46%) (Table 1).

**Table 1:** Baseline characteristics of the patients.

Variables	CAS (≥50%)		No CAS (<50%)		All patients	
	n=36		n=130		n=166	
	n	%	n	%	n	%
Mean age (years±SD) (min-max)	68.66±9.46		63.04±9.55		64.25±9.78	
Male gender	29 (80.55)		101 (77.69)		130 (78.31)	
DM	16 (44.44)		49 (37.69)		65 (39.15)	
Hypertension	30 (83.33)		96 (73.84)		126 (75.90)	
Dyslipidemia	19 (52.77)		68 (52.30)		87 (52.41)	
BMI (mean±SD)	26.83±2.92		26.90±3.38		26.88±3.27	
History of PAD	6 (16.66)		11 (8.46)		17 (10.24)	
Smoking	27 (75.00)		96 (73.84)		123 (74.09)	
History of CVA	5 (13.88)		2 (1.53)		7 (4.22)	
Left ventricular ejection fraction	61.22±8.84		61.75±9.91		61.63±9.66	
ACCT (min)	75.50±18.46		76.11±23.66		75.98±22.58	
TPT (min)	116.31±20.56		117.34±28.72		117.12±27.11	
Number of graft vessels	3.86±0.99		3.41±0.92		3.51±0.95	
LMCA disease	10 (27.77)		24 (18.46)		34 (20.48)	

ACCT=aortic cross-clamp time; BMI=body mass index; DM=diabetes mellitus; LMCA=left main coronary artery; PAD=peripheral arterial diseases; TPT=total perfusion time

Logistic regression analysis revealed that the model was compatible and significant according to risk factors (omnibus chi-square=24.052, *P*=0.013). In addition, in the model, 11 variables explained 21% of CAS development in patients who underwent CABG (Negelkerke R square=0.208). Older age (OR=1.064, 95% CI%=1.015-1.116, *P*=0.011) and a history of CVA (OR=0.135, 95% CI%=0.021-0.869, *P*=0.035) increased the risk of CAS development (Table 2). There were no significant differences in terms of gender (*P*=0.632), DM (*P*=0.984), HT (*P*=0.274), dyslipidemia (*P*=0.431), BMI (*P*=0.938), smoking (*P*=0.585), history of PAD (*P*=0.595), number of graft vessels (*P*=0.081), and LMCA disease (*P*=0.433) (Table 2).

**Table 2:** Results of risk factors for significant carotid stenosis (≥50% luminal narrowing).

Factors	B	SE	*P*	Odds ratio	95% CI
Age	0.062	0.024	0.011	1.064	1.015-1.116
Gender	-0.352	0.735	0.632	0.703	0.167-2.968
DM	-0.009	0.438	0.984	0.991	0.420-2.339
Hypertension	-0.636	0.582	0.274	0.529	0.169-1.656
Dyslipidemia	-0.354	0.450	0.431	0.702	0.290-1.695
BMI	0.005	0.069	0.938	1.005	0.879-1.150
History of PAD	-0.356	0.671	0.595	0.700	0.188-2.608
Smoking	0.369	0.674	0.585	1.446	0.386-5.423
History of CVA	-2.003	0.950	0.035	0.135	0.021-0.869
Number of graft vessels	0.424	0.243	0.081	1.527	0.949-2.457
LMCA disease	-0.396	0.504	0.433	0.673	0.250-1.809

BMI=body mass index; CVA=cerebrovascular accident; DM=diabetes mellitus; LMCA=left main coronary artery; PAD=peripheral arterial disease

The overall incidence of CVA after CABG was 1.20% (n=2). Postoperative CVA rate was 5.55% (n=2) in the stenosis group, and no CVAs were observed in the group without stenosis. CAS increased the risk of postoperative CVA after CABG (omnibus chi-square=6.203, *P*=0.013). None of the patients died from complications due to postoperative CVA. Preoperative evaluation of the two patients who developed CVA revealed that one patient had 60% stenosis in the left ICA and the other had 30% stenosis in the left ICA and 60% stenosis in the right ICA. During the early postoperative period, ipsilateral hemiplegia developed in one (0.60%) patient, and this patient had a history of CVA 6 months ago. CVA (motor aphasia and quadriplegia) developed on the 6^th^ postoperative day in the other patient. These patients with major CVA were discharged with neurological sequelae at the 2^nd^ and 3^rd^ postoperative months after treatment at the neurology clinic. In the follow-up program for CAS, 28 patients with moderate stenosis in any of the carotid arteries and one patient with asymptomatic 70% stenosis and no CEA were enrolled.

## DISCUSSION

Cerebrovascular complication is the one of the most dreadful complications after CABG, with a reported incidence of 2.1 to 5.2%, and results in acute mortality of up to 38%^[11-13]^. In patients planned to undergo CABG, the rate of severe CAS is 6% (range 3.2-8.7%)^[14-16]^. In the present study, the prevalence of severe CAS was 4.81% (n=8). Two of these eight patients had total occlusion. Patients with heart problems that have been resolved but who have had a CVA during the postoperative period remain mostly bedbound at the end of prolonged hospitalization periods and are also frequently lost. In a comprehensive study, the 30-day CVA risk after CABG was 1.1%^[17]^. In their study on 1499 patients undergoing cardiac surgery, Adams et al.^[18]^ found the rate of perioperative CVA of 1.73% (26 patients). Carotid disease is an important etiological factor in the pathophysiology of CVA after CABG. However, even assuming that prophylactic carotid endarterectomy carried no additional risk, it could prevent only approximately 40 to 50% of procedural CVAs^[19]^. Although CVA after CABG is multifactorial, CAS may be the cause of CVA through various mechanisms. Carotid intraplaque hemorrhage can result in plaque destabilization and intimal ulceration, creating a nidus for thromboembolism. Anticoagulating patients during CABG might be responsible for the increased intraoperative risk of intraplaque hemorrhage. Mechanical causes can also trigger intraplaque hemorrhage, such as turbulent blood flow and hypertension, which can occur during cardiac surgery. Impaired cerebral hemodynamic function distal to CAS is another determinant of CVA post-CABG^[19]^. In our study, the prevalence of CVA after CABG was 1.20% (n=2) within 30 days. This can be explained by the identification of patients with severe CAS in the preoperative period and the performance of CEA.

The fact that patients who are planned for CABG and who have severe CAS are largely asymptomatic during the preoperative period creates difficulties in the diagnosis^[20]^. Therefore, the comorbidity of coronary and carotid artery disease should be evaluated in detail during the preoperative period. Khan et al.^[21]^ reported that color Doppler sonography is nowadays the first imaging examination performed for the diagnosis of carotid artery stenosis. Its dual ability to evaluate both morphologic and hemodynamic abnormalities and its cost-effectiveness make color Doppler ultrasound the only test applied before a therapeutic decision^[21]^.

Some studies have examined the necessity of screening the carotid artery system of patients with DUS during the preparation stage before CABG. Okur et al.^[4]^ recommended that coronary artery patients aged <65 years old should also be routinely screened by DUS in the preoperative evaluation, regardless of risk factors. Using preoperative carotid DUS, Cornily et al.^[22]^ found that the rate of severe CAS was 5.8% (n=12) in 205 consecutive patients undergoing CABG. They also identified severe CAS as a risk factor for postoperative CVA and recommended selective screening with carotid DUS in patients aged >70 years old, with carotid bruit, with a history of cerebrovascular disease, DM or PAD. In 2018, the European Society of Cardiology/European Association for Cardio-Thoracic Surgery (ESC/EACTS) Guidelines recommended carotid DUS in patients aged ≥70 years old with no history of CVA/TIA in the last 6 months (class IIb, level of evidence B)^[23]^. In our study, the mean age of eight patients with ≥70% stenosis was 66.00±11.94 (range 54-82) years, and 50% (n=4) of these eight patients were <70 years old. If we had performed selective carotid DUS in patients aged ≥70 years old, these four patients would not have been detected. In their study with 3708 patients who underwent open heart surgery, Ascher et al.^[24]^ found that the prevalence of significant carotid disease was >4.5% in patients aged >60 years old. In that study, routine carotid DUS was recommended for patients aged >60 years old, regardless of the accompanying risk factors, whereas carotid DUS was recommended in patients aged <60 years old, when there were at least two major risk factors such as hypertension, DM, and smoking.

In their study on 722 cardiac surgery patients, of whom 36.3% had coronary artery disease, Chun et al.^[25]^ identified independent risk factors for CAS as peripheral vascular disease after a previous CVA and coronary artery disease with left main or three-vessel disease. Anastasiadis et al.^[2]^ found that a history of CVA and the presence of bruit on clinical examination were significant predictors of severe carotid disease. In our study, age and history of CVA were identified as risk factors for CAS. PAD is strongly associated with CAS. Preoperative screening with carotid DUS provides valuable information on asymptomatic CAS and identifies patients with severe asymptomatic CAS who are at a high risk of CVA to consider more intensive management of carotid disease in PAD patients^[26]^. In our study, 10.24% (n=17) patients had PAD, and PAD was not identified as a risk factor for CAS. CAS rate is higher in patients with LMCA disease^[27]^. In our study, 20.48% (n=34) of patients undergoing CABG had LMCA disease, but LMCA disease was not a risk factor for CAS.

Carotid angiography is a gold standard method for the diagnosis of carotid artery disease. However, carotid DUS is more frequently used because it is non-invasive and easily applicable in clinical practice. Taneja et al.^[28]^ recommend the incorporation of this commonly available and easy-to-use bedside technique of Doppler examination of the carotid vessels in routine intraoperative practice as the standard of care in all patients undergoing CABG^[28]^. In our clinic, carotid DUS screening is performed routinely regardless of risk factors in all patients undergoing CABG. Carotid artery system angiography is performed in patients with ≥70% stenosis detected by DUS, and treatment options are determined according to the symptomatic condition of the patient. We consider it important to identify patients with moderate CAS during the post-CABG period and to enroll these patients in follow-up programs. In our study, 16.86% (n=28) of 166 CABG patients with carotid DUS had moderate stenosis in either carotid artery. With the addition of one patient who did not undergo CEA, a total of 29 patients were enrolled in the follow-up program for CAS.

## CONCLUSION

Routine carotid DUS method aims to reduce the risk of postoperative CVA caused by severe CAS in patients undergoing CABG. At the same time, indirect CVA risks, such as determining the location of central venous catheterization and necessity of not reducing the pump pressure, are also reduced by identifying CAS in patients, even when it is not at the surgical margin. In addition, identifying and monitoring patients with moderate CAS during the postoperative period will help reduce the risk of late CVA in these patients.

In conclusion, this study demonstrated that age and a history of CVA are independent risk factors for CAS in CABG patients. We recommend carotid screening for all patients undergoing CABG patients, regardless of the absence of associated risk factors.

**Table:** 

Authors' roles & responsibilities	
HK	Substantial contributions to the conception or design of the work; or the acquisition, analysis, or interpretation of data for the work; drafting the work or revising it critically for important intellectual content; final approval of the version to be published
